# Ligand-Induced Movements of Inner Transmembrane Helices of Glut1 Revealed by Chemical Cross-Linking of Di-Cysteine Mutants

**DOI:** 10.1371/journal.pone.0031412

**Published:** 2012-02-20

**Authors:** Mike Mueckler, Carol Makepeace

**Affiliations:** The Department of Cell Biology and Physiology, Washington University School of Medicine, St. Louis, Missouri, United States of America; University of Cambridge, United Kingdom

## Abstract

The relative orientation and proximity of the pseudo-symmetrical inner transmembrane helical pairs 5/8 and 2/11 of Glut1 were analyzed by chemical cross-linking of di-cysteine mutants. Thirteen functional di-cysteine mutants were created from a C-less Glut1 reporter construct containing cysteine substitutions in helices 5 and 8 or helices 2 and 11. The mutants were expressed in Xenopus oocytes and the sensitivity of each mutant to intramolecular cross-linking by two homobifunctional thiol-specific reagents was ascertained by protease cleavage followed by immunoblot analysis. Five of 9 mutants with cysteine residues predicted to lie in close proximity to each other were susceptible to cross-linking by one or both reagents. None of 4 mutants with cysteine substitutions predicted to lie on opposite faces of their respective helices was susceptible to cross-linking. Additionally, the cross-linking of a di-cysteine pair (A70C/M420C, helices 2/11) predicted to lie near the exoplasmic face of the membrane was stimulated by ethylidene glucose, a non-transported glucose analog that preferentially binds to the exofacial substrate-binding site, suggesting that the binding of this ligand stimulates the closure of helices at the exoplasmic face of the membrane. In contrast, the cross-linking of a second di-cysteine pair (T158C/L325, helices 5/8), predicted to lie near the cytoplasmic face of the membrane, was stimulated by cytochalasin B, a glucose transport inhibitor that competitively inhibits substrate efflux, suggesting that this compound recruits the transporter to a conformational state in which closure of inner helices occurs at the cytoplasmic face of the membrane. This observation provides a structural explanation for the competitive inhibition of substrate efflux by cytochalasin B. These data indicate that the binding of competitive inhibitors of glucose efflux or influx induce occluded states in the transporter in which substrate is excluded from the exofacial or endofacial binding site.

## Introduction

The passive exchange of glucose across the membranes of animal cells is mediated by members of the GLUT (SLC2a) protein family (reviewed in [1], [2], [3]). The GLUT family belongs to the Major Facilitator Superfamily (MFS), the largest category of proteins involved in the transport of small molecules across membranes [4], [5]. Glut1, the prototype member of the GLUT family and the first eukaryotic member of the MFS Superfamily to be identified and cloned [6], [7], is one of the most extensively studied of all membrane transporters [8]. Kinetic and biophysical studies of glucose transport in the human red blood cell are mostly consistent with an alternating conformation mechanism [9], [10], [11], [12], [13] but see [12], [14]), a conclusion that is consistent with high-resolution structural studies of 4 bacterial MFS proteins [15], [16], [17], [18].

Glut1 was the first transporter predicted to possess 12 transmembrane helices [7], a feature that it appears to share with the vast majority of MFS transporters [4]. This prediction has been confirmed by glycosylation-scanning mutagenesis experiments [19] and other biochemical analyses (reviewed in [20]). The 12 transmembrane helix model for Glut1 is also strongly supported by the deduced structures of the lac permease [15], the glycerol-3-P antiporter [17], the fucose transporter [16], and the EmrD multidrug transporter [18], all members of the MFS expressed in E. coli. These 4 bacterial transporters share a common folding pattern, despite the fact that they share little if any sequence identity. Several of the twelve proposed transmembrane segments of Glut1 were originally predicted to form amphipathic alpha-helices, an observation which led to the hypothesis that these helices form the walls of a water-filled cavity involved in the binding and subsequent transfer of glucose across the membrane [7]. It was also suggested that hydroxyl- and amide-containing amino acid side chains within the transmembrane helices form the sugar-binding site of Glut1 via hydrogen bonding with glucose hydroxyl groups.

Considerable experimental support has accumulated for this basic structural model. Cysteine-scanning mutagenesis and substituted cysteine accessibility studies implicate transmembrane segments 1 [21], 2 [22], 5 [23], 7 [22], [24], 8 [25], 10 [26], and 11 [27] of Glut1 in the formation of a water-accessible cleft within the membrane. In contrast, helices 3 [28], 6 [29], 9 [30], and 12 [31] appear to have limited access to the external solvent, suggesting that these segments form the outer stabilizing helices as indicated by the known bacterial MFS structures [15], [16], [17], [18]. Transmembrane segment 4 of Glut1 does not appear to react with pCMBS added to the external solvent [32]. This transmembrane segment is predicted to be an inner helix in the outward-facing conformation of the fucose transporter, indicating that one face should be accessible to the external solvent [16]. Thus, either the two structures differ or reaction of helix 4 with pCMBS cannot be detected in Glut1 for structural reasons that are unclear at present. Gln^161^ within helix 5 [33] and Gln^282^ within helix 7 [34] appear to participate in forming the exofacial substrate-binding site. Val^165^, which is positioned one helical turn distant from Glu^161^, is accessible to aqueous sulfhydryl reagents and appears to lie near the exofacial substrate binding site based on mutagenesis and inhibitor studies [35]. An aromatic side-chain at position 412 within helix 11 appears to be essential for transport activity [20]. Additionally, hydrogen exchange studies demonstrate that 30% of peptide hydrogen atoms are exposed to water in purified, reconstituted Glut1, consistent with their role in the formation of an aqueous cleft in the membrane [36]. Little is known, however, about movements of specific helices in Glut1 that occur during the transport cycle or after the binding of ligands.

In the present study we utilized chemical cross-linking of di-cysteine (di-C) Glut1 mutants constructed in a reporter molecule to determine the relative orientation and proximity of transmembrane helices 5/8 and 2/11. Both pairs of helices are predicted to comprise a part of the inner helical bundle that forms the outward-facing aqueous cavity [30] and appear to lie within ∼6 Å of each other throughout much of their lengths, suggesting that they lie roughly parallel to each other in at least one conformational state. Additionally, the binding of a non-transported exofacial ligand, ethylidene glucose, promoted the closure of helices 2 and 11 at the exoplasmic face of the membrane. In contrast, cytochalasin B recruited Glut1 to a conformation in which helices 5 and 8 close at the cytoplasmic face of the membrane. This latter observation provides the first data concerning the relative movement of specific helices and pairs of amino acid residues of Glut1 induced by cytochalasin B binding, and provides a possible explanation for the competitive inhibition of substrate efflux by cytochalasin B.

## Results

A reporter Glut1 molecule (C-TEV) was engineered in order to facilitate the determination of the relative proximity and orientation of pairs of transmembrane helices using chemical cross-linking of di-cysteine (di-C) mutants, an experimental approach that has been successfully used to analyze the structure of many integral membrane proteins [37]. A TEV protease recognition site was introduced into the large, central, cytoplasmic loop of C-less Glut1 (Glut1 whose 6 native cysteine residues were mutated to threonine or serine residues), permitting the analysis of pairs of cysteine residues residing in opposite halves of the molecule after chemical cross-linking. We have previously demonstrated that C-less Glut1 exhibits close to wild-type transport activity when expressed in Xenopus oocytes [35]. The single site of N-linked glycosylation was also eliminated in the reporter Glut1 construct in order to simplify quantification of the data by preventing the smearing of the bands on SDS gels due to heterogeneous glycosylation. The 2-deoxyglucose transport activity of the resultant C-TEV construct expressed in oocytes was ∼40% of the activity of the parental C-less Glut1 protein (0.33 versus 0.82 pMoles/oocyte/30 min/unit protein expression).

The transmembrane helical pairs 2/11 and 5/8 are predicted to lie adjacent to one another and to form half of the inner bundle of helices that form the outward-facing aqueous substrate-binding cavity [30]. In order to further test this model and to detect movements of inner transmembrane helices during ligand binding, 13 Glut1 mutants were constructed in which cysteine residues were substituted into each pair of helices (see Table 1). All of the paired cysteine substitutions were placed at positions predicted to lie at roughly the same location along the axis perpendicular to the plane of the membrane according to the original topological model [7]. However, 9 of the 13 substitutions were chosen so that the paired cysteine residues were predicted to lie directly across from one another in their respective helices, whereas the other four dicysteine mutants were made in paired residues predicted to be positioned on opposite faces of their helices. We would thus predict that at least some of the group of 9 dicysteine mutants should be subjected to intramolecular chemical cross-linking via their sulfhydryl groups, whereas none of the 4 control mutants should be cross-linked.

**Table 1:** Construction of di-cysteine mutants in a C-Less GLUT 1 reporter molecule.

	Helix 2	Helix 11
A70C/M420C	GCC→TGC	ATG→TGC
V74C/T413C	GTT→TGT	ACC→TGC
G76C/N411C	GGC→TGC	AAC→TGC
M77C/F409C	ATG→TGC	TTC→TGC
F81C/A405C	TTC→TGC	GCC→TGC
L85C/A402C	CTT→TGT	GCT→TGT
F86C/I404C	TTC→TGC	ATT→TGT

	Helix 5	Helix 8
L156C/V327C	CTG→TGC	GTG→TGC
T158C/L325C	ACC→TGC	CTG→TGC
V165C/T321C	GTC→TGC	ACT→TGC
G172C/G314C	CAG→TGT	GGT→TGT
F174C/A309C	TTC→TGC	GCC→TGC
L176C/V307C	CTG→TGC	GTG→TGC

An aglyco Glut1 reporter molecule was created by mutating the consensus site of N-linked glycosylation at N45 to a threonine residue and by inserting a Tev protease cleavage site (ENLYFQG) between residues 247 and 248 in the central cytoplasmic loop of Glut1. This reporter construct was then used to make the above 13 dicysteine mutants in helices 2/11 and helices 5/8.

**Figure 1:**
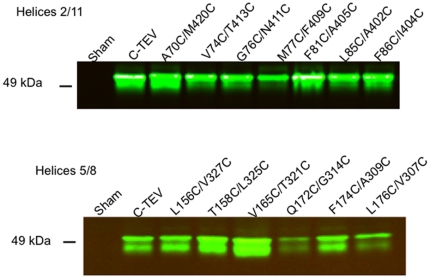
Expression of dicysteine mutants in Xenopus oocyte membranes. Stage 5 *Xenopus* oocytes were injected with water (Sham) or with 50 ng of mRNA encoding the parental reporter construct (C-Tev) or the indicated dicysteine mutant. Two days post injection total oocyte membranes were prepared and subjected to immunoblot analysis using a rabbit polyclonal ab raised against a peptide corresponding to the C-terminal 15 residues of human Glut1.

**Figure 2:**
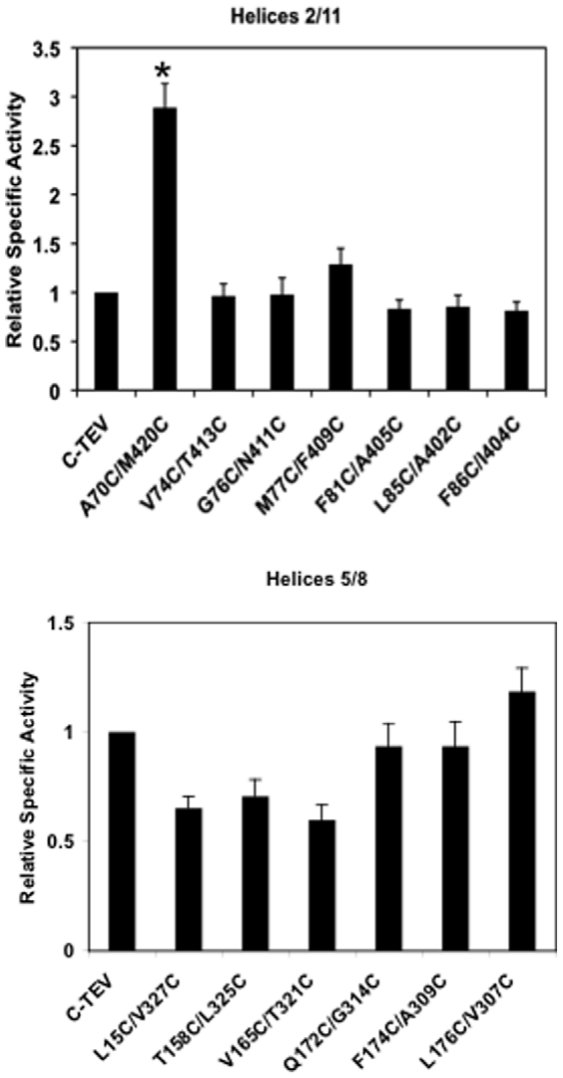
2-Deoxyglucose uptake activity of Glut1 mutants. [^3^H]-2-DOG uptake (50 µM, 30 min. at 22°C) was measured 2 days after injection of oocytes with 50 ng of mRNA. Activities were normalized to the value measured for oocytes expressing the control C-Tev construct (0.18±0.03 pMoles/oocyte/30 min/unit band intensity). Unit band intensity refers to the relative intensity of the protein bands as measured by immunoblot analysis using a Li-Cor imager and normalizes for varying levels of protein expression for the different mutants. Results represent the mean ± SE of 3–6 independent experiments with 15–20 oocytes per experimental group. Values observed in water-injected oocytes were subtracted.

**Figure 3:**
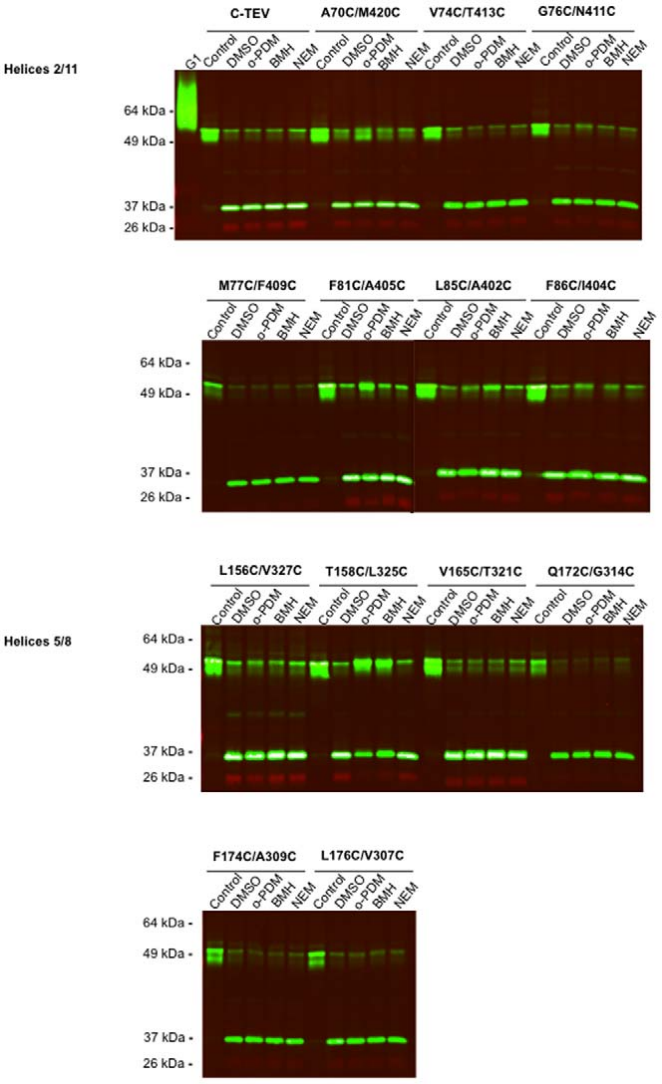
Chemical Cross-linking of di-C Mutants. Stage 5 *Xenopus* oocytes were injected with 50 ng of mRNA encoding the parental reporter construct (C-Tev) or the indicated dicysteine mutants (see Table 1). After incubation of oocytes for 2 days, cross-linking analysis was conducted on purified oocyte membranes as described in “Materials and Methods”. The reactions were quenched by the addition of 2 mM cysteine and oocyte membranes were digested with Tev protease then subjected to SDS-PAGE followed by immunoblotting with rabbit polylclonal ab raised against the C-terminal 15 residues of human Glut1 (red bands) and a mouse monoclonal ab that recognizes an epitope in the N-terminal half of the central cytoplasmic loop (green bands). Note that the full-length ∼54 kD bands were recognized by both antibodies and show up as yellow when the intensity of the detector was increased. “C-TEV” is the control cysteine-less parental construct. “Control” lanes were loaded with membranes that were not subjected to either chemical cross-linking or protease cleavage. “DMSO” lanes were loaded with membranes that were not subjected to chemical cross-linking but were digested with TEV protease. The “o-PDM” and “BMH” lanes were loaded with membranes that were subjected to cross-linking by the respective chemical and then were treated with TEV protease. The ratio of the intensities of the full-length bands in the DMSO lanes to those in the Control lanes thus provide the maximum level of protease cleavage for each mutant. The ratio of the intensities of the full-length bands in the “o-PDM” or ‘BMH” lanes to those in the Control lanes indicate the extent of cross-linking by either reagent. This ratio is termed the cross-linking efficiency or “fraction cross-linked” in Table 2.

Figure 1 shows an immunoblot demonstrating that all 13 dicysteine mutants were efficiently expressed in Xenopus oocyte membranes. The mutant proteins appeared as doublets and the relative intensity of the two members of the doublet varied in some of the mutants relative to the parental construct. Whether the doublet is the result of the existence of distinct conformers of the proteins under the conditions of SDS-PAGE or the presence of an unknown partial post-translational modification is not known. However, the existence of the doublets did not influence the results of the cross-linking experiments described below, nor did the relative ratio of the doublet members correlate with transport activity. All of the mutants exhibited 2-deoxyglucose uptake activities in intact oocytes that were comparable to the parental C-Tev control, except for A70C/M420C, which showed a nearly 3-fold increase in transport activity (Figure 2). Altered transport activity, either enhancement or inhibition, has been observed for many Glut1 point mutants (see [30] and references therein). The results of preliminary experiments involving time courses and concentration curves indicated that maximal cross-linking of dicysteine mutants was achieved when membranes were incubated for 20 min in the presence of 1.0 mM BMH (bis-maleimidehexane), a flexible sulfhydryl-specific cross-linker ∼16 Å in length, or 1.0 mM o-PDM (1,4-Phenylenedimaleimide), a relatively rigid molecule ∼6 Å in length [38] (data not shown). After the 20 min incubation period, cross-linking was quenched by the addition of 5 mM cysteine, the membranes were digested with TEV protease, and then subjected to immunoblot analysis. If the two cysteine residues in a dicysteine mutant are in the proper orientation and close enough to one another in proximity, the mutant transporter should show pseudo-protease resistance proportional to the efficiency of the cross-linking reaction. Figure 3 shows a representative series of immunoblots obtained for all 13 mutants as well as the control parental C-Tev construct. Cleavage of the full-length ∼54 kD control construct (C-Tev, upper panel), which lacks cysteine residues, generated an N-terminal fragment of ∼32 kDa (green bands) and a C-terminal fragment of ∼26 kDa (red bands). Cleavage was ∼90% efficient. Cross-linking of the mutants is indicated by a reduction in the disappearance of the full-length ∼54 kD bands along with a corresponding decrease in the appearance of the N- and C-terminal fragments relative to samples incubated with vehicle alone (lanes labeled “DMSO”) prior to protease cleavage. The DMSO lanes thus provided an estimate of the maximum protease cleavage efficiency for each mutant. Samples were also treated with 1.0 mM NEM to determine whether modification of sulfhydryl groups alone in the absence of cross-linking affected protease cleavage (lanes labeled “NEM”). NEM did not affect the protease cleavage of any of the dicysteine mutants.

Quantification of the results of 3–8 independent experiments is presented in Table 2. The data are expressed as the intensity of the full-length protein bands present in the protease-digested cross-linked samples (“o-PDM”, “BMH” lanes in Figure 3) divided by the intensity of the full-length bands present in the undigested control samples (“Control” lanes in Figure 3). These values are denoted as the cross-linking efficiencies in Table 2. Notably, none of the 4 dicysteine mutants with substitutions at residues predicted to lie on opposite faces of their respective helices exhibited cross-linking, whereas 5 of the 9 mutants with substitutions at residues predicted to lie in direct apposition to one another were subject to statistically significant levels of cross-linking by at least one of the reagents. The highest levels of cross-linking were observed for mutants A70C/M420C and F81C/A405C (helices 2/11) and T158C/L325C (helices 5/8). Much weaker but statistically significant levels of cross-linking were also observed for L85C/I404C (helices 2/11) and Q172C/G314/C (helices 5/8). Each of these 5 mutants was susceptible to cross-linking by o-PDM, whereas only T158C/L325C and A70C/M420C were cross-linked by BMH. NEM had no effect on the protease cleavage of any of the 13 mutants. Control experiments indicated that none of the dicysteine mutants were subjected to inter-molecular cross-linking, as noted by the absence of oligomers when samples were analyzed on non-reducing gels (data not shown). These data indicate that these 5 sets of paired residues lie within ∼6–16 Å of each other at some point during the transport cycle.

**Table 2:** Cross-linking Efficiency of Glut1 Dicysteine Mutants.

	Fraction Cross-Linked							
Double Mutant	1.0 mM o-PDM				1.0 mM BMH			
	Average	SE	n	p value	Average	SE	n	p value
**Helices 2/11**								
A70C/M420C	0.266	0.098	5	0.001	0.056	0.032	5	0.169
V74C/T413C	−0.070	0.029	3	0.071	−0.054	0.025	3	0.096
G76C/N411C	−0.109	0.072	3	0.204	−0.221	0.130	3	0.163
M77C/F409C	−0.047	0.048	3	0.387	−0.060	0.040	3	0.208
F81C/A405C	0.307	0.100	5	0.001	−0.004	0.037	5	0.488
L85C/A402C	0.080	0.029	5	0.002	0.094	0.035	5	0.005
F86C/I404C	0.006	0.050	3	0.911	0.029	0.081	3	0.741
**Helices 5/8**								
L156C/V327C	−0.036	0.016	4	0.039	−0.071	0.028	5	0.033
T158C/L325C	0.750	0.112	6	0.001	0.464	0.065	7	0.001
V165C/T321C	−0.025	0.014	7	0.073	−0.013	0.017	8	0.445
G172C/G314C	0.050	0.019	6	0.019	0.019	0.045	7	0.703
F174C/A309C	0.011	0.025	4	0.670	−0.057	0.023	5	0.065
L176C/V307C	−0.018	0.040	4	0.669	−0.027	0.029	5	0.433

Oocyte membranes expressing dicysteine mutants were subjected to chemical cross-linking as described in Materials and Methods. Cross-linking efficiency is expressed as the intensity of the full-length transporter bands in the cross-linked lanes (o-PDM and BMH lanes, see Figure 3) divided by the intensity of the full-length transporter bands in the control lanes, after subtraction of background intensities. See Figure 3 for a representative set of immunoblots. The results of 3–8 independent experiments are presented.

Previous protease sensitivity experiments have suggested that significant conformational changes accompany the binding of ligands to Glut1 [39], [40]. However, no studies have yet been reported concerning movements that may occur in specific pairs of transmembrane helices or paired amino acid residues of a glucose transporter during the transport cycle or after ligand binding. In order to determine whether helices 5/8 and 2/11 undergo relative displacement after ligand binding, two inhibitors of glucose transport were used. Cytochalasin B competitively inhibits sugar efflux and non-competitively inhibits sugar uptake [41], [42]. This observation suggests that the binding of glucose at the endofacial substrate-binding site and of cytochalasin B to its binding site on Glut1 are mutually exclusive events, although it does not indicate where on the Glut1 molecule cytochalasin B binds. Ethylidene glucose is a non transported substrate analog that competitively inhibits substrate influx, most likely by binding preferentially to the exofacial binding site of Glut1 [43].

Because the covalent cross-linking reactions would eventually reach a plateau regardless of any competitive effect of the reversible binding of the two transport inhibitors, early time points were examined using lower concentrations of o-PDM and BMH in order to observe whether the inhibitors had any effect on the cross-linking reactions. Concentrations and time points were based on preliminary experiments for each mutant that maximized the observed effects.

**Figure 4:**
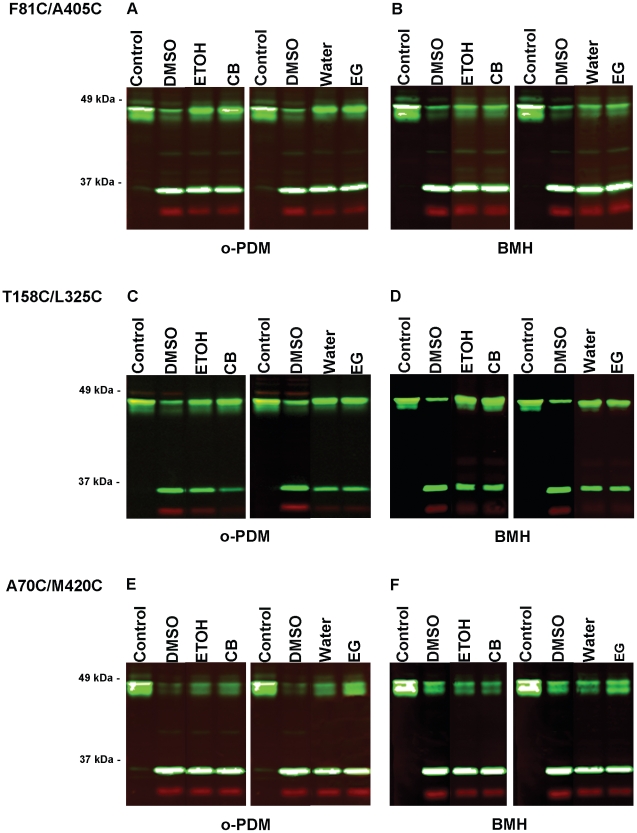
Effect of non-transported ligands on chemical cross-linking. Oocyte membranes were incubated in presence of either vehicle alone (water or ethanol) or 50 µM cytochalasin B or 50 mM ethylidene glucose for 5 min prior to the addition of the indicated concentration of either o-PDM or BMH. Cross-linking efficiency was measured by protease cleavage followed by immunoblot analysis as described in “Materials and Methods”. The water lanes represent the controls for the addition of ethylidene glucose and the ethanol lanes controlled for the addition of cytochalasin B. The DMSO lanes represent samples to which DMSO was added but no cross-linker. These lanes indicate the maximum cleavage observed for each mutant with TEV protease.

**Figure 5:**
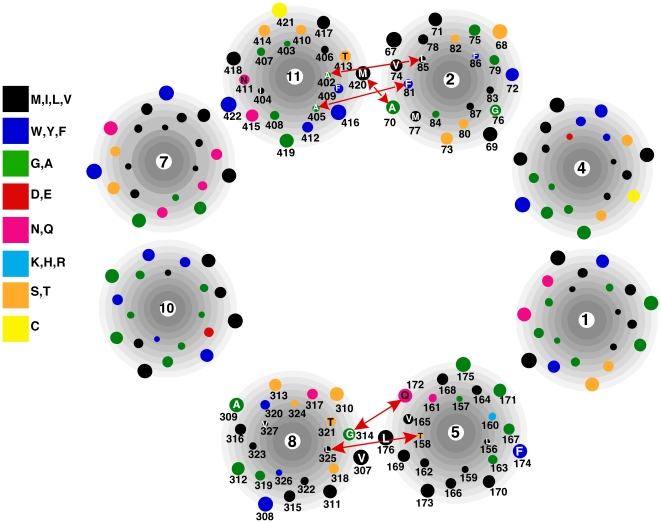
Cross-sectional model of the inner transmembrane helices of Glut1 in the exoplasmic conformation as viewed from the exoplasmic face of the membrane based on experimental results. Amino acid residues subjected to cysteine substitution in the dicysteine mutants are given by the single letter code. Red lines connect residues that were cross-linked by o-PDM or BMH within the helical pairs 2/11 and 5/8.

**Figure 6:**
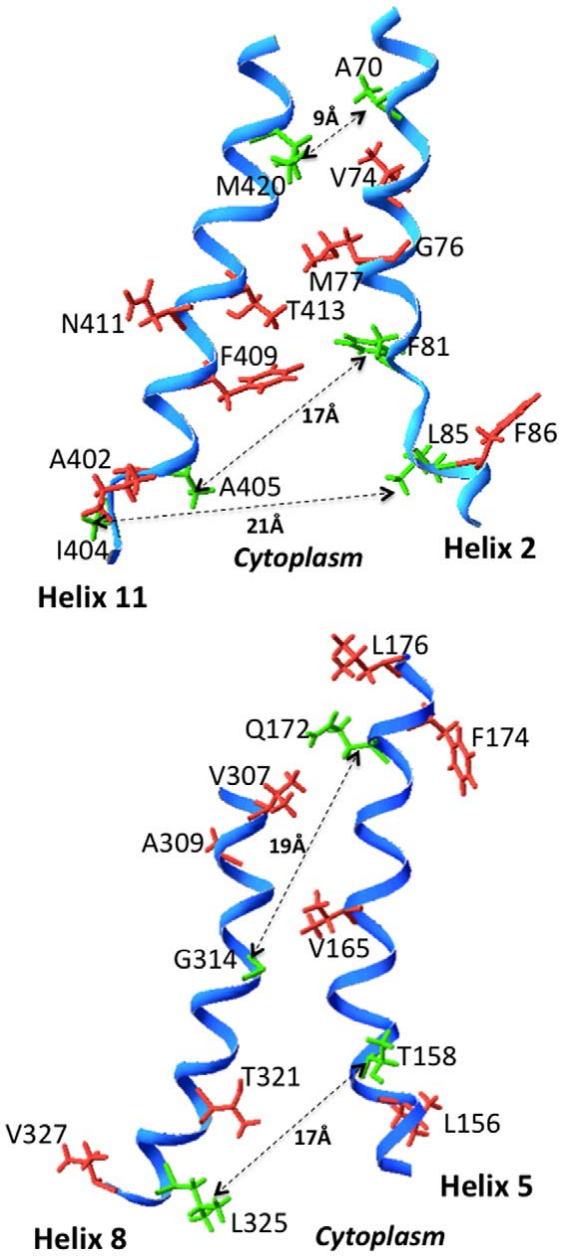
Side-view of the orientation of helices 2/11 and 5/8 of Glut1 in the endofacial conformation based on homology modeling. The orientation of the helices is derived from an homology-based model of Glut1 that used the structure of the E. coli Glycerol-3-P Antiporter as the template molecule [17]. Side chains of residues that were mutated to cysteines and subjected to chemical cross-linking analysis are identified by their single letter amino acid codes. Residues that exhibited cross-linking are shown in green in ball and stick form and are connected by dotted lines. Distances between cross-linked residues are given in angstroms (Å). Residues that did not exhibit cross-linking are shown in red.

The effects of cytochalasin B and ethylidene glucose on the kinetics of cross-linking of 3 of the 5 susceptible dicysteine mutants are shown in Figure 4 and quantification of the results is presented in Table 3. The cross-linking of L85C/A402C and Q172C/G314C could not be accurately assessed by this analysis because of their low levels of maximal cross-linking (5% and 8%, respectively, see Table 2). Neither cytochalasin B nor ethylidene glucose had any significant affect on the cross-linking of F81C/A405C (Figures 4A and B). Interestingly, however, cytochalasin B stimulated the cross-linking of T158C/L325C by o-PDM by 1.8 fold (P = 0.02) but had no affect on cross-linking by BMH (Figures 4C and D). Ethylidene glucose had no effect on either cross-linking reaction. In contrast, ethylidene glucose enhanced the cross-linking of A70C/M20C by BMH by 10-fold (P = 0.03), whereas cytochalasin B had no significant affect on cross-linking (Figure 4E and F). Ethylidene glucose also stimulated the cross-linking of A70C/M420C by o-PDM by 2-fold, but this effect was not statistically significant. These data demonstrate that the binding of a non-transported substrate analog to the exofacial binding site or the binding of a competitive inhibitor of substrate efflux induce conformational changes in Glut1 that result in the relative movements of the inner helical pairs 2/11 or 5/8, respectively. T158C/L325C has cysteine substitutions at the cytoplasmic ends of helices 5 and 8, whereas A70C/M20C has cysteine substitutions at the exoplasmic ends of helices 2 and 11 (see Figures 5 and 6). Thus, these results indicate that cytochalasin B recruits Glut1 to a conformational state whereby the cytoplasmic ends of helices 5 and 8 move closer together, and that ethylidene glucose promotes a conformational state in which the exoplasmic ends of helices 2 and 11 move closer together.

**Table 3:** Effect of Ligands on the Cross-Linking of Glut1 Di-cysteine Mutants.

Double Mutant	0.1 mM o-PDM				0.1 mM BMH			
	Average	SE	n	p value	Average	SE	n	p value
**F81C/A405C**								
ETOH	0.347	0.104	4		0.099	0.090	4	
CB	0.440	0.143	4	0.620	−0.002	0.050	4	0.336
Water	0.334	0.102	4		−0.027	0.037	4	
EG	0.320	0.085	4	0.917	0.002	0.054	4	0.674

T158C/L325C	0.3 mM o-PDM				0.3 mM BMH			
ETOH	0.273	0.041	5		0.609	0.175	4	
CB	0.489	0.062	5	0.019	0.804	0.230	4	0.556
Water	0.448	0.081	5		0.697	0.114	4	
EG	0.437	0.100	5	0.938	0.632	0.106	4	0.784

A70C/M420C	0.1 mM o-PDM				0.1 mM BMH			
ETOH	0.048	0.059	4		0.001	0.069	4	
CB	0.037	0.070	4	0.916	−0.076	0.040	4	0.373
Water	0.118	0.044	4		0.017	0.038	4	
EG	0.270	0.079	4	0.143	0.199	0.048	4	0.026

Oocyte membranes expressing dicysteine mutants were subjected to chemical cross-linking as described in Materials and Methods in the presence or absence of cytochalasin B (CB) or ethylidene glucose (EG). Cross-linking efficiency is expressed as the intensity of the full-length transporter bands in the cross-linked lanes (o-PDM and BMH lanes, see Figure 4) divided by the intensity of the full-length transporter bands in the control lanes, after subtraction of background intensities. See Figure 4 for a representative set of immunoblots. The results of 4–5 independent experiments are presented. Ethanol (ETOH) is the control for the addition of cytochalasin B (CB), and Water is the control for the addition of ethylidene glucose (EG).

## Discussion

A minimum of 30 percent sequence identity is required in order to accurately model a membrane protein structure based on homology [44]. Mammalian glucose transporters do not share more than ∼10 percent sequence identity with any of the 4 bacterial transporters of the MFS whose structures have been deduced by x-ray diffraction, nor do they appear to share the minimum 30 percent sequence identity with any known prokaryotic proteins. Thus, other approaches to exploring the structure of these proteins are required to make progress in this area in the absence of x-ray diffraction data. Additionally, alternate biochemical approaches to structure are usually complementary to, and not precluded by, x-ray data.

The data presented in this study demonstrate that 5 of 13 dicysteine mutant Glut1 transporters were susceptible to intramolecular chemical cross-linking by homobifunctional, thiol-specific cross-linking reagents. Cross-linking was observed between two pairs of predicted inner transmembrane helices, 2/11 and 5/8. The cross-linking data are consistent with a model for the structure of Glut1 based on a comprehensive series of scanning mutagenesis studies employing the substituted cysteine accessibility method [21], [22], [23], [24], [26], [27], [28], [29], [30], [31], [32] as well as previous cross-linking studies [45] and with the overall protein folding pattern observed for bacterial members of the MFS. All 5 of the susceptible dicysteine mutants exhibited cross-linking by o-PDM and 2 of the mutants were cross-linked by both o-PDM and BMH. o-PDM and BMH have linkers that permit cross-linking distances of up to ∼6 Å and ∼16 Å, respectively [38]. However, successful cross-linking by a specific reagent also depends on the precise spatial orientation and accessibility of the reactive groups. Cross-linking of both pairs of helices in the absence of ligand occurred at residues predicted to lie near the exoplasmic and cytoplasmic faces of the membrane, indicating that at some point or points during the transport cycle one or both ends of these pairs of helices lie within ∼6 Å of each other, consistent with the close proximity predicted by the 2-dimensional model shown in Figure 5. The fractional cross-linking efficiency of 2 of the mutants (L85C/A402C, Q172C/G314C) was very low, although still statistically significant. Whether this low efficiency was due to steric and geometrical constraints, the accessibility of the homobifunctional reagents to these sulfhydryl groups, or to some other factor, cannot be determined at present.

The cross-linking of 3 of the mutants allowed us to evaluate the effect of non-transported ligands on cross-linking behavior and to draw inferences concerning the basic conformational states induced by these inhibitors. Cytochalasin B, a mold metabolite, is one of the most potent low molecular weight inhibitors of Glut1 activity that has been reported [46]. Several different observations have led to the assumption that cytochalasin B binds to the endofacial conformation of the transporter, possibly overlapping the substrate-binding site, and thus inhibiting activity: First, cytochalasin B is a competitive inhibitor of substrate efflux and a non-competitive inhibitor of substrate influx [41]. Second, homology modeling of Glut1 structure suggested a cytoplasmic docking site for cytochalasin B [47]. Third, the kinetics of dissociation of the cytochalasin B/Glut1 complex stimulated by an exofacial ligand are consistent with cytochalasin B binding to the endofacial conformation [48]. Fourth, modeling studies suggest that cytochalasin B may bind to a substrate-binding site of the transporter via hydrogen bonds and hydrophobic interactions analogous to those that occur between glucose and Glut1 [49]. These observations strongly support the view that cytochalasin B binds to Glut1 in its endofacial configuration, but do not provide direct evidence as to the sidedness or precise location of cytochalasin B binding on Glut1. Mutagenesis experiments indicate that W^388^ and W^412^ are involved in the Glut1/cytochalasin B interaction [50], [51], and protease digestion experiments suggest that photolabeling of Glut1 by cytochalasin B involves a ∼3 kD region encompassing these two tryptophan residues [52].

In the present study cytochalasin B stimulated the cross-linking of residues T158C and L325C. These residues are predicted to lie near the cytoplasmic ends of helices 5 and 8, respectively (see Figure 6). This indicates that cytochalasin B binding decreases the distance between these two residues and suggests that cytochalasin B binding promotes closure of the helical bundle near the cytoplasmic face of the membrane. Recent evidence suggests that Glut1 may possess distinct high and low affinity binding sites for cytochalasin B [42]. When the higher affinity site is occupied, transport activity is stimulated, but the occupation of the lower affinity site at higher ligand concentrations results in transport inhibition. Cytochalasin B was present at saturating inhibitory concentrations during the cross-linking reactions. We speculate that cytochalasin B-induced closure of helices near the cytoplasmic face of the membrane prevents access of glucose to the endofacial substrate-binding site, which would be observed as competitive inhibition of substrate efflux [41], [53], [54]. Cytochalasin B might induce closure of the helices by binding to the endofacial substrate-binding site or to an allosteric binding site on the Glut1 molecule. The only requirement is that the binding promotes a structural change in Glut1 that induces sufficient closure of the endofacial cavity to prevent access to the substrate-binding site, and binding must occur to Glut1 in the endofacial conformation. The present data do not have any implications as to whether cytochalasin B binds to an exofacial region, endofacial region, or intramembranous domain of Glut1.

4,6-O-ethylidene glucose is a Glut1 substrate analog that appears to bind to the exofacial substrate binding site with ∼10-fold greater apparent affinity compared to the endofacial site [41], [43], [54]. The presence of high concentrations of this modified sugar should thus initially recruit Glut1 to the exofacial conformation. An interesting question is what happens after this non-transported ligand binds to the exofacial site? Crystal structures of MFS proteins in the endofacial [15], [17] and exofacial [16] conformations reveal that inner transmembrane helices splay open to allow access to a substrate binding site at one side of the membrane and pinch together at the opposite side of the membrane to seal off the aqueous cavity. From these diffraction data it might be predicted that ethylidene glucose would, at least initially, recruit Glut1 molecules to the outward configuration with the inner transmembrane helices spread apart at the exoplasmic side of the membrane. In apparent contradiction to this prediction, our data indicate that the presence of ethylidene glucose dramatically enhanced the cross-linking of residues A70C and M420C in a dicysteine mutant by up to ∼20-fold (see Figure 4), suggesting that binding of the sugar analog to the exofacial site causes helices 2 and 11 to pinch together. The simplest interpretation of these data is that after the initial binding of ethylidene glucose to the exofacial site, the transporter undergoes a conformational change in which the exoplasmic ends of the inner helices close around the bound ligand, thus decreasing the average distance between residues A70C and M420C and increasing the degree of cross-linking. The bulky ethylidene group between C-4 and C-6 may prevent the full conversation of the transporter to the endofacial configuration, so that the transporter is transiently present in an intermediate conformation before the complex relaxes back to the exofacial configuration followed by release of the bound ligand. Alternatively, the transporter may fully convert to the exofacial conformation but fail to release the ligand because of the ethylidene moiety.

An alternative interpretation for the closure of helices 5 and 8 or helices 2 and 11 after cytochalasin B or ethylidene glucose binding, respectively, is that the pinching together of these helices is required for the opening of other inner transmembrane helices that is in turn required to allow access to the cytoplasmic or exoplasmic substrate binding site.

The results of the cross-linking experiments reported herein are completely consistent with the low resolution 2-dimensional model of the relative orientation of the inner transmembrane helices in the Glut1 exofacial conformation deduced from a comprehensive series of mutagenesis and solvent accessibility experiments (summarized in [30], see Figure 5). Thus, all of the observed cross-linking may in theory have occurred with Glut1 in the open exofacial conformation or in an alternative conformation where the relative positions of the cross-linked residues do not differ significantly from that in the exofacial conformation. In an attempt to assess the possibility that one or more cross-linking events may have occurred with Glut1 in the endofacial conformation, we examined the orientation and distance between cross-linked residues in a homology-based model of Glut1 [47] (see Figure 6). The distance between the cross-linked residues in the model varies from ∼9 to ∼21 Å. Given that all five cross-linking events were observed with o-PDM, which allows a maximum distance of 6–8 Å between residues [38], it appears that either none of the cross-links occurred in the endofacial configuration of the transporter, or alternatively, the model is not accurate. The ability of cytochalasin B to enhance the cross-linking of T158C and L325C strongly suggests that this event occurred with the transporter in the endofacial configuration (see the above discussion). This implies that the homology-based model is not accurate with respect to the orientation of helices 5 and 8. It should be mentioned that the glycerol-3-P antiporter used as the template in the homology modeling shares little if any sequence identity with Glut1, and that a minimum of ∼30% identity is required between two proteins for accurate homology-based modeling [44].

The data presented in the study provide evidence that the binding of non-transported ligands to Glut1 promotes movements of inner transmembrane helices and support an alternating conformation-type transport mechanism [55], consistent with the comparative structures of bacterial MFS proteins. However, elucidation of the glucose transport mechanism ultimately is dependent on the crystallization of the transporter in multiple configurations in the presence and absence of substrate.

## Materials and Methods

### Ethics Statement

All experiments involving Xenopus frogs were conducted with the approval of the Washington University Animal Studies Committee (Protocol #200110049).

### Materials

*Xenopus laevis* –Imported African frogs were purchased from Xenopus Express (Homosassa, FL), ^3^H-2-deoxyglucose and Diguanosine triphosphate (mRNA cap) were purchased from Amersham Pharmacia Biotech (Arlington Heights, IL), Megascript™ RNA synthesis kit was purchased from Ambion Inc (Austin, TX), Transformer™ Site-Directed mutagenesis kit was obtained from Clontech (Palo Alto, CA). SuperSignal TM West Pico Chemiluminiscent Substrate and Bismaleimidehexane (BMH) were obtained from Pierce (Rockford, IL), 1,4-Phenylenedimaleimide (o-PDM) was purchased from Aldrich Chemical Co. (Milwaukee, WI), and Decylmaltoside (DM) was obtained from Anatrace Inc. (Maumee, OH).

### General Procedures

Procedures for the site-directed mutagenesis and sequencing of human Glut1 cDNA and the in vitro transcription and purification of Glut1 mRNAs (25), isolation, microinjection, and incubation of *Xenopus* oocytes (26), preparation of oocyte membranes (23), SDS polyacrylamide gel electrophoresis and immunoblotting with Glut1 C-terminal antibody (20), and 2-deoxyglucose uptake measurements (27), have been described in detail previously.

### Construction of di-Cysteine Glut1 Mutants

C-less Glut1 cDNA [23], [56] subcloned into the oocyte expression vector pSP64T was subjected to site-directed mutagenesis to produce an aglyco C-less Glut1 containing the amino acid motif for a Tev protease cleavage site(Glu-Asn-Leu-Tyr-Phe-Gln-Gly) within the large, central cytoplasmic loop between residues Gly^247^ and S^248^. The site of N-linked glycosylation was mutated (Asn^45^ to Ser) in order to simplify quantification of the protein bands (see Table 1). This construct, designated C-Tev, was then used as a template to create 13 di-cysteine mutants in the helical pairs 5/8 and 2/11 (see Table 1). The cysteine substitutions were generated extending from the inner face to the outer face of the membrane in each of the helices.

### Treatment with homobifunctional maleimide cross-linking reagents and effect of transport inhibitors

Stage 5 *Xenopus* oocytes were injected with 50 ng of each mutant Glut1 mRNA. Two days after injection, total membranes were prepared from groups of 15–20 oocytes. Three micrograms of freshly isolated total membranes from injected oocytes were incubated with the indicated concentrations of o-PDM, BMH, or NEM in 50 mM Tris-HCl pH 7.4, 0.5 mM EDTA, 1 mM dithiothreitol for various time periods at 22°C. In the inhibitor experiments either cytochalasin B (50 µM) or ethylidene glucose (50 mM) were added to the samples 5 min prior to the addition of the cross-linking reagents. Control experiments demonstrated that up to 50 mM L-glucose had no effect on the cross-linking reactions (data not shown). The reaction was quenched by the addition of 2 mM cysteine and the membranes were then treated with 10 units of TEV protease (In Vitrogen) for 1 h at 22°C. The digested membranes were then analyzed by SDS PAGE and immunoblot analysis using a rabbit polyclonal ab raised against a peptide corresponding to the C-terminal 15 residues of human Glut1 (1∶10,000 dilution) and a mouse monoclonal ab that recognizes a motif within the N-terminal half of the central cytoplasmic loop (1∶10,000 dilution of ascites fluid). The secondary abs used were LiCor donkey anti-mouse IRDye 800 CW and donkey anti-rabbit IRDye 680 (both at 1∶10,000 dilution) in conjunction with LiCor blocking buffer. Imaging of the blots was conducted using a LiCor Odyssey infrared imaging system model 9120 and system version 3.0.

### Statistical Analysis

Data were analyzed for statistical significance using the two-tailed, unpaired, Student's T-test.
